# Cost-effective 3D scanning and printing technologies for outer ear reconstruction: current status

**DOI:** 10.1186/s13005-023-00394-x

**Published:** 2023-10-27

**Authors:** György Wersényi, Verena Scheper, Simone Spagnol, Thomas Eixelberger, Thomas Wittenberg

**Affiliations:** 1 Széchenyi István University, Győr, H-9026 Hungary; 2 Department of Otolaryngology, Hannover Medical School, Hannover, D-30625 Germany; 3 Universitá Iuav di Venezia, Venice, I-30135 Italy; 4 Friedrich-Alexander-University Erlangen-Nuremberg & Fraunhofer Institute for Integrated Circuits IIS, Erlangen, D-91058 Germany

**Keywords:** 3D scanning and reconstruction, 3D printing, Volumetric scanning, Additive manufacturing, Clinical application, Patient-centered medicine, Patient-individualized therapy, Outer ear

## Abstract

Current 3D scanning and printing technologies offer not only state-of-the-art developments in the field of medical imaging and bio-engineering, but also cost and time effective solutions for surgical reconstruction procedures. Besides tissue engineering, where living cells are used, bio-compatible polymers or synthetic resin can be applied. The combination of 3D handheld scanning devices or volumetric imaging, (open-source) image processing packages, and 3D printers form a complete workflow chain that is capable of effective rapid prototyping of outer ear replicas. This paper reviews current possibilities and latest use cases for 3D-scanning, data processing and printing of outer ear replicas with a focus on low-cost solutions for rehabilitation engineering.

## Introduction

Rehabilitation engineering is the development of technological solutions and devices to assist individuals with disabilities, targeting the recovery of physical and/or cognitive functions lost because of disease or injury [1–3] (https://www.nibib.nih.gov/science-education/science-topics/rehabilitation-engineering). Engineers design and build devices, applications, and systems to meet a wide range of needs that can assist individuals and help people with their daily activities (work, exercise, education), specifically focusing on aesthetic issues, functionality, and safety. In healthcare, the term reconstruction engineering refers to activities related to reconstruction of damages to the body, usually of the bones [4]. Tissue engineering focuses on development and production of living human tissue [5–7] (https://www.todayonline.com/world/surgeons-transplant-3d-ear-made-living-cells-1915476). The goal is to assemble functional constructs to restore, maintain or improve damaged tissues or whole organs. Good examples of this are artificial skin and cartilage.

During rapid prototyping (RP), the fabrication of a physical object or assembly is achieved during a fast manufacturing process with the help of 3D printing technology. The creation is usually completed using layered additive manufacturing (3D printing), but other conventional technologies (i.e., molding, extruding) can be applied as well [8, 9]. RP allows for individually adjusted personalized fabrication of items in the short run, including the use of biomaterials [10–13].

In recent years, 3D printing has gained momentum in rehabilitation since printers and materials have become commercially available and affordable, and this includes pre-clinical rehabilitation engineering [14, 15]. However, these 3D printed materials are not based on (living) cells and tissue. Dentistry – addressing hard, bony tissue in the human body – was the first medical area that implemented 3D-printed implants, followed by orthopedics [16–20]. Another prominent example of reconstructive *hard, bony tissue* manufacturing is from the field of maxillofacial surgery and relates to the additive manufacturing of the orbita [21]. Cost savings based on 3D hard-tissue printing in clinical applications were explored in various clinical disciplines, including general surgery, maxillofacial surgery and orthopedics, and was even considered for radiology [22–28]. It allows for personalized manufacturing instead of mass production [28–31].

However, these 3D printed constructs are per se imitating bone, and their stiffness is therefore generally tremendously high. Only a few patient cases using flexible material exist [32]. To our knowledge, reconstructions based on (living) cells and tissue are still at laboratory stage. Fast and atraumatic scanning of the area of interest is a perquisite for clinical acceptance of this approach. The printed part must be elastic to ensure smooth attachment to the rest of the tissue and a physiological appearance.

## Fundamentals

This paper reviews actual trends and developments in outer ear 3D replication technology, including scanning, image processing and printing, focusing on cost effective reconstruction of the outer ears, and highlighting pros and cons of the different technologies. To this end, within this section, the basic workflow (from scanning to printing) will be introduced (“Basic workflow” section) as well as a brief introduction to the outer ear anatomy will be provided (“Outer ear anatomy” section).

### Basic workflow

**Fig. 1:**
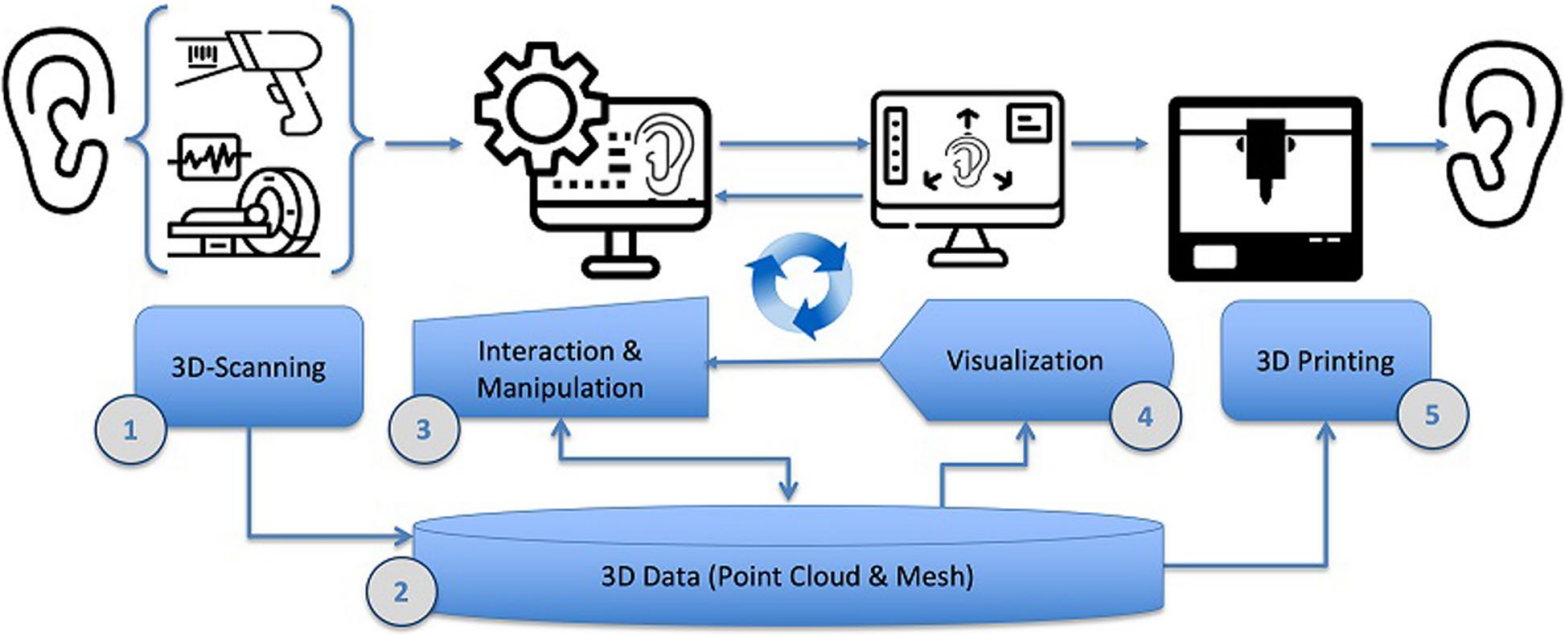
Workflow pipeline for rapid prototyping of outer ear replicas. From left to right: (1) 3D-scanning (“3D data acquisition possibilities for the outer ear” section), either volumetric (“Optical 3D-scanning and capturing devices” section) or optical (“Optical 3D-scanning and capturing devices” section); (2) repository of scanned 3D data (local or cloud-based); (3) interactive correction, manipulation and enhancement of the 3D data (“Data processing” section); (4) interactive 3D-visualization and inspection of the ear data; (5) additive 3D manufacturing of the ear replica (“3D printing” section). Steps (2), (3) and (4) are tightly coupled in a closed-loop, as the 3D visual inspection and 3D correction generally go hand-in-hand

Figure 1 shows a general workflow pipeline of 3D replication of outer ear structures. The workflow starts with 3D data acquisition (see “3D data acquisition possibilities for the outer ear” section) using various types of optical or volumetric MRT/CT scanning devices, which yields 3D-data that is stored in a local or online (cloud-based) data repository (1, 2). The next steps are 3D image processing and interactive manipulation (see “Data processing” section) for image correction and post-processing (3), and interactive 3D-visualisation (4). The final step consists of 3D-printing (5) (see “3D printing” section). Note, that steps (2), (3), and (4) are tightly coupled in a closed-loop manner, as the established 3D point cloud data (2) can be manipulated and corrected (3), and visualized and inspected (4) from different views. Simultaneously, this workflow, coarsely depicted in Fig. 1, serves as a guideline through this paper.

**Fig. 2:**
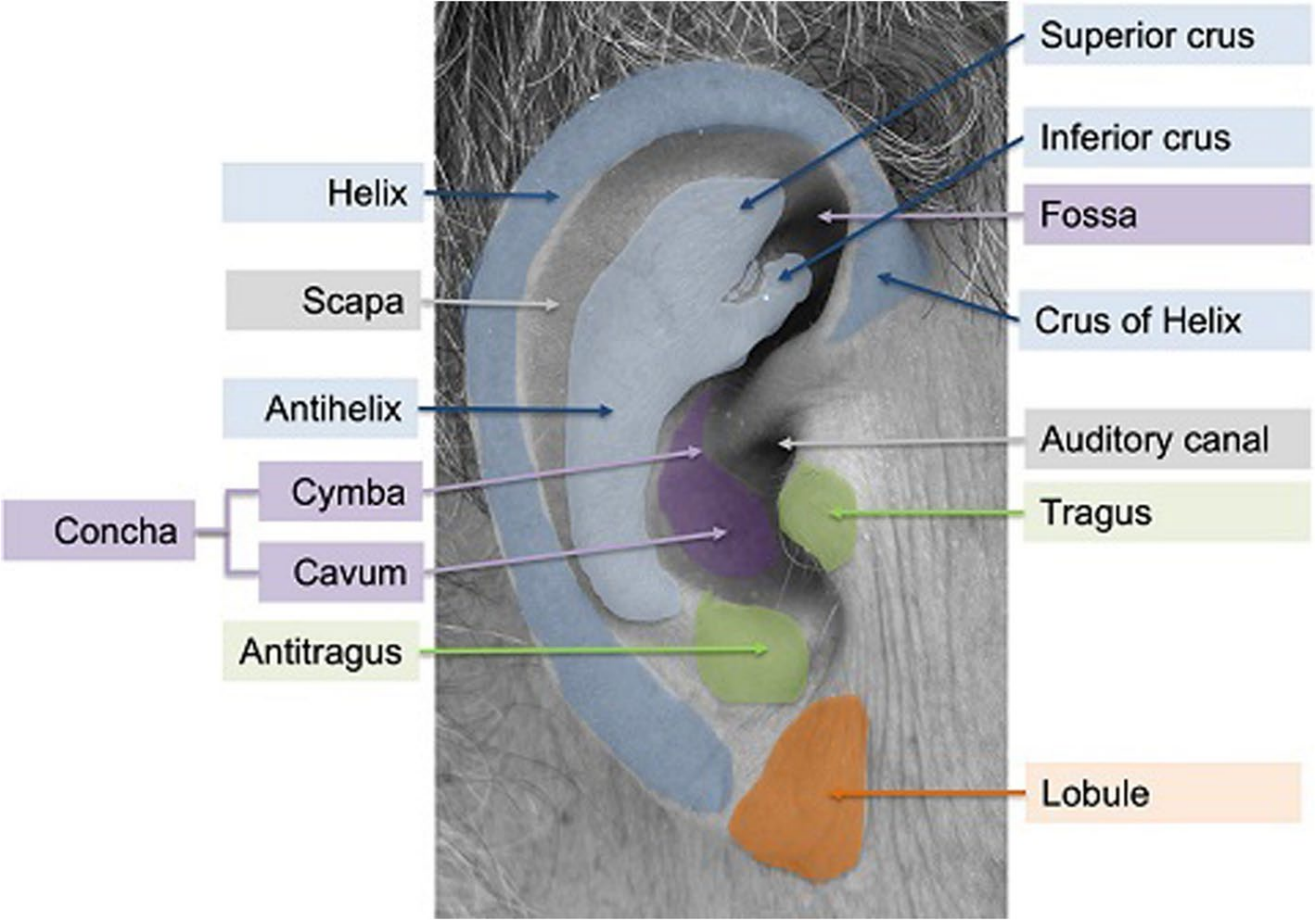
Anatomy of the outer / external right human ear (of author TW), consisting (from the inside out) of the external end of auditory canal in the center (gray), surrounded by *tragus* and *antitragus* (green), the *antihelix* and *helix* with their ‘legs’ (*crus*) on the top (in blue), which enclose the *concha* and *fossa* (purple), and the *scapa* (gray). The bottom part of the ear is the ‘lobe’ (orange)

Currently, several – academic and commercial – workflow pipelines have been adapted and optimized for outer ear replicas [33–39]. These applications focus on cost effectiveness based on estimated price/piece values, time expenditure and quality of end-products. The main motivation is to make the technology affordable and available for the wider public by keeping costs low for patients and/or insurance companies. The outer ear – also referred to as the ‘auricle’ or ‘pinna’ (cf. “Outer ear anatomy” section and Fig. 2) – is one of the most addressed and tested body parts in terms of low effort personalized prosthetic 3D printing. As an outer and generally not moving body part, requirements for the (re)production pipeline can be lower than for other body parts. Avoiding growing tissue, invasive surgery or sterilization reduces costs and makes the related procedures faster and more effective. However, this technology also has flaws and limitations.

### Outer ear anatomy

For a visual orientation and reference, the anatomic structure of the outer ear (the auricle) is displayed and described in Fig. 2. From inside out, the external ear consists of the end of the auditory canal in the center (gray), which is directly surrounded by the *tragus* and *antitragus* (green). The main geometry of the ear is formed by the *antihelix* and *helix* with their ‘legs’ (‘*crus*’) on the top (in blue), which enclose the *concha* and *fossa triangle* (purple). The bottom part of the ear is the ‘lobe’ (orange).

## 3D data acquisition possibilities for the outer ear

To obtain adequate 3D data from human ear lobes to serve as production files for additive manufactured *outer ear* reproduction, two main approaches for image data acquisition can be considered. From the clinical side, volumetric data (see “Volumetric data acquisition” section) from magnetic resonance tomography imaging (MRI) [27], computer tomography (CT) [40–42] or cone beam computer tomography (CBCT) is readily available, and can be integrated in the clinical routine without difficulty.

Nevertheless, from an economical point of view, handheld 3D scanning devices (see “Optical 3D-scanning and capturing devices” section) are less costly, easy-to-use for non-radiologists, and yield similar results as that of volumetric scanning systems.

Both scanning approaches – volumetric as well as some optical approaches – are also used to obtain image data about the external auditory canal to personalize the earmolds of hearing aids and to improve diagnosis and therapy planning of middle ear (with *incus, stapes* and *malleus*, the semicircular canals and the cochlea) pathologies. In contrast to these special applications, in our contribution we only focus on the acquisition and reproduction of the anatomy of the *outer* or *external* ear.

### Volumetric data acquisition

Traditional volumetric or MRI imaging [43] is usually performed to obtain detailed internal images of the soft tissue of the human body. A computed tomography (CT) scan is a volumetric imaging technique via a rotating X-ray tube and detectors to measure the X-ray attenuation of different tissues. CT scans are usually applied if hard tissue is involved, such as the human skull [40–42]. Moreover, contrary to full-body scanners, digital volume tomography (DVT) can be relatively small and are available in increasing numbers in ENT clinics [44–46].

**Fig. 3:**
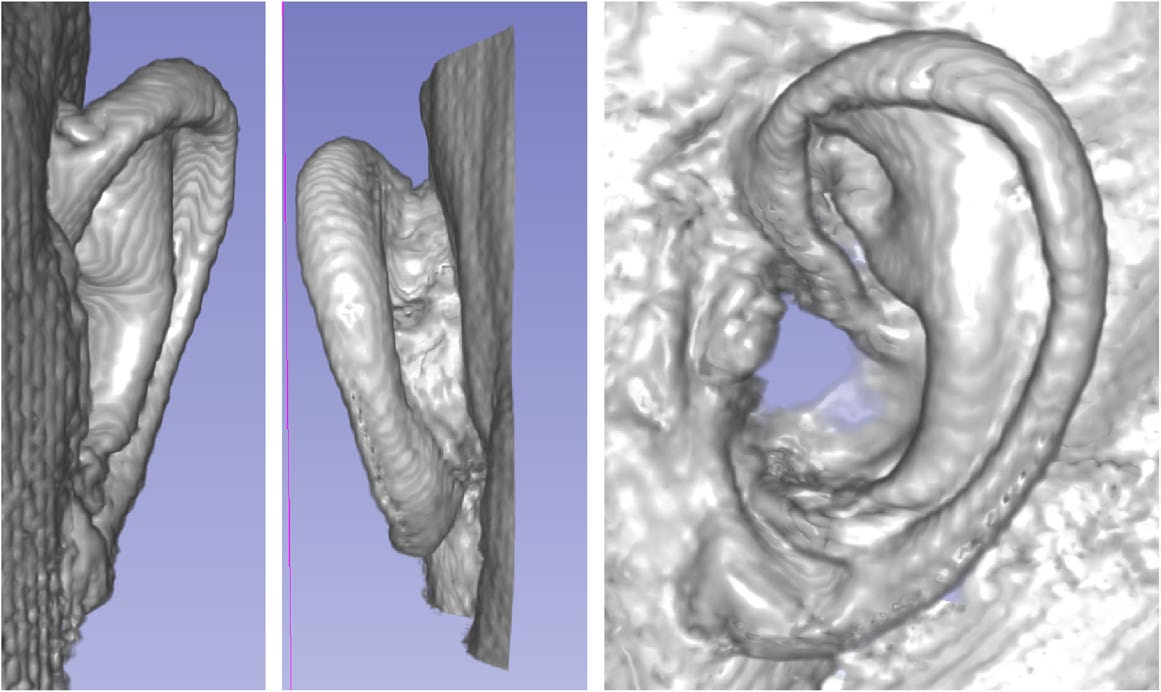
Examples of a 3D reconstruction of a left outer ear from a 3T MRI scan (of author TW) using Slicer 3D software [47]. Left side: posterior-anterior view; center: anterior-posterior view; right side: side-view

In contrast to computed tomography (CT or DVT), which applies X-ray sources, magnetic resonance imaging (MRI) makes use of strong magnetic fields, magnetic field gradients, and radio waves to generate images of the organs in the body. Compared to CT and DVT, MRI provides better soft tissue contrast. However, patients find MRIs less comfortable due to usually longer and louder measurements and the subject’s placement is in a tight, confining head coil, which is then placed in the tube (the gantry). Additionally, implants, such as pace makers or artificial joints and other non-removable metallic objects in the human body may pose a risk and exclude some patients from undergoing an MRI examination safely. In addition, the introduced volumetric image-capturing approaches are mainly limited to hospital and clinical use. As an example of MRI data of the outer ear, Fig. 3 depicts a left ear (of author TW) in posterior-anterior view (left), anterior-posterior view (center), and side view (right) using Slicer software for 3D-volume rendering [47].

All three image modalities yield volumetric image data, and therefore, can be used to build 3D models. The volumetric data, usually packed in the widely used DICOM standard file format has to be converted to STL format for further processing [48, 49]. This type of data is the basis for additive manufacturing of patient-individualized objects or devices.

### Optical 3D-scanning and capturing devices

An alternative to volumetric scanners is optical scanners used in combination with 3D reconstruction possibilities (“photogrammetry”) for 3D-surface and scene reconstruction. To this end, the surface of 3D objects in the real world can be scanned with different technologies. These technologies include, for example, line-scanning [50], structured-light scanners [51–53], LiDAR systems, and Time Of Flight (TOF) scanners, which are all capable of detecting and mapping shapes and forms of 3D-objects in the real world to three dimensional models [54, 55]. The field of biomedical engineering has adapted various scanning methods, which includes those for facial scans [51, 52, 56–58]. Furthermore, these scanning technologies can be differentiated in triangulation systems (either having a camera and a light source, or two cameras) or purely monocular systems where reconstruction is obtained in a successive step.

**Fig. 4:**
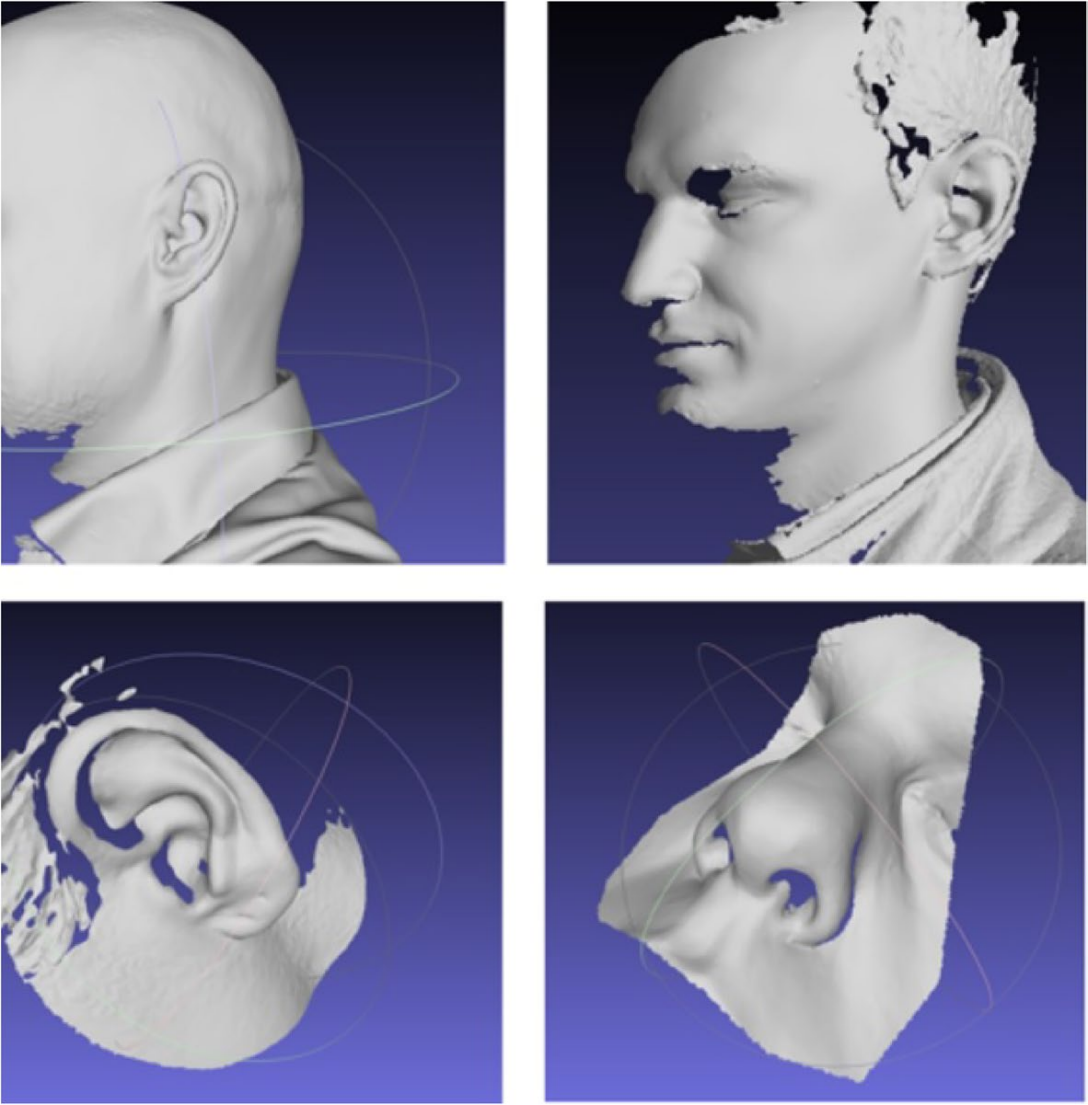
Examples of facial scans using handheld scanners (Go!SCAN, top row; SIMSCAN 3D, bottom row) for post-processing in MeshLab

Recently, handheld scanning devices have become popular due to their easy accessibility, user-friendly handling, and pricing [38, 59–63]. During triangulation, a laser dot or line is projected onto an object from the device while it measures the distance to the surface. The scanner is in motion, so a position of it has to be constantly updated by applying references on the surface or by external tracking methods. Figure 4 shows examples of facial images using handheld scanners. Similarly, stereo systems can also be used [64].

In contrast to expensive and large 3D scanning devices, handheld scanners have the advantage of being small in size, portable, inexpensive, and easy to use. Some of the models currently commercially available have recently been evaluated for facial scans, both for ears and nose [38]. Accuracy and user friendliness were found to be fulfilling with respect to the requirements of further data processing. Tested models apply a LED or laser light for scanning, along with output formats compatible with image processing software and that have a spatial resolution for detailed printed models in the price range of 10 to 30 thousand Euro.

Also most recently, monocular scanning devices – such as smartphones or digital (SLR) cameras – combined with computer vision approaches have yielded promising results for the assessment and 3D reconstruction of the outer ear. This idea is supported by a recent comprehensive survey showing that for the case of outer ears, the data obtained with commercial 2D imaging devices can be enhanced by 3D reconstruction [65]. Furthermore, the geometric shapes of 3D ears can be used to improve recognition accuracy.

**Fig. 5:**
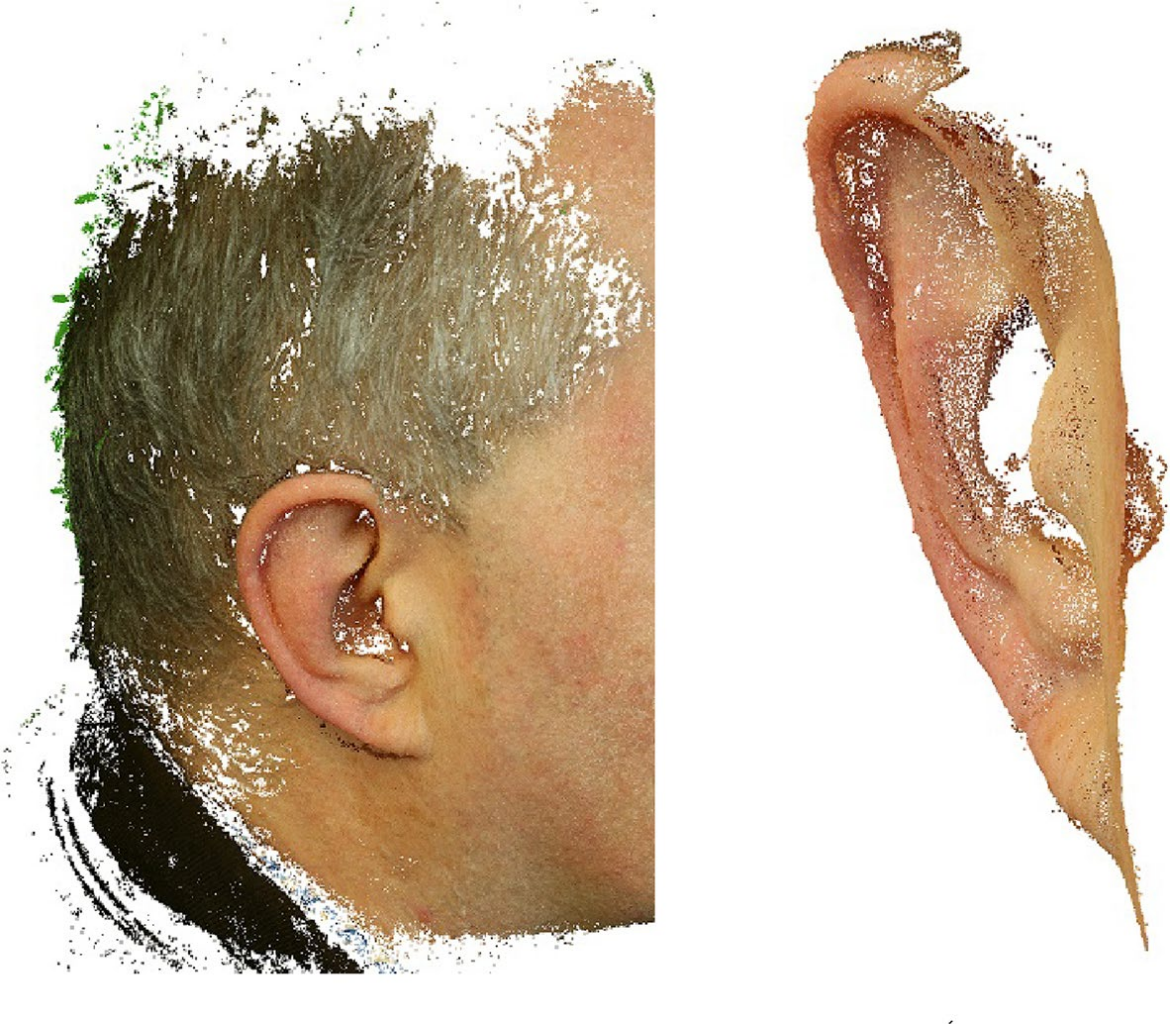
3D Scanning and reconstruction of the pinna (of author TW) using a digital SLR camera and open-source reconstruction software. The structures of the *helix*, *antihelix*, and *scapa* are reconstructed correctly, while the dimples of the *concha* and *fossa* remain as white spots

**Fig. 6:**
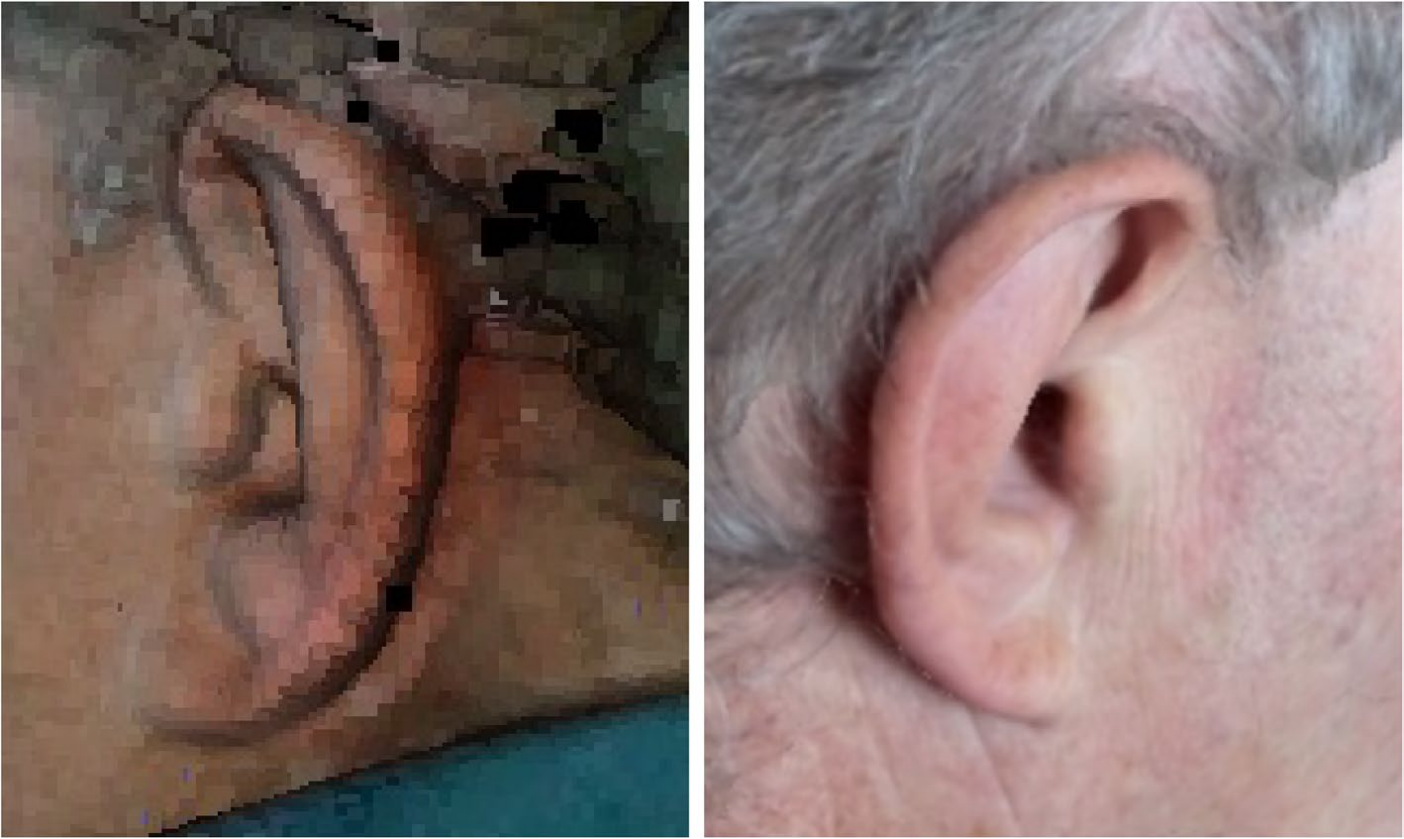
3D Scanning and reconstruction of the pinna (of author TW) using a commercial handheld 3D scanning device (3DSense) (left), and a smartphone (right) in combination with an open-source reconstruction software (PolyCam)

Figure 5 shows an example of an outer ear scan and 3D reconstruction using a portable digital SLR camera and open-source reconstruction software [66, 67]. With respect to the outer ear anatomy, in this example, it can be observed that the structures of the *helix* and *antihelix* with the *scapa* could be reconstructed quite well, while the dimples of the *concha* and *fossa* are depicted as non-reconstructed white spots. Figure 6 provides two examples of an outer ear scan and 3D reconstruction using a (a) low-cost commercial handheld 3D line scanning device (3DSense) (left) and (b) the other was obtained from a smartphone (iPhone 13) (right) and post-processed with an open-source reconstruction software (PolyCam). In both examples the 3D reconstruction software achieved good results for the outer ear anatomy and its texture, including even the dimples of the *concha* and *fossa*.

Even though optical large-scale 3D-scanners [68, 69] have also recently become available for full body scanning in medical applications [70, 71], we restrict our focus in this field to cost-efficient, high-throughput, handheld optical 3D scanning approaches of the outer ear only. For applications, millimeter accuracy is required, but most of the scanners offer even sub-millimeter scanning accuracy and mesh resolution.

A table of some investigated handheld optical 3D-scanning devices can found in Appendix A (Table 1).

## Data processing

### Software interfaces

One component placed between the (optical or volumetric) scanner and the acquired image data is the so-called *software interface* and is usually provided by the vendor of the scanning devices. It can also be considered as a tri-lateral interface between (a) the hardware of the scanning-device (driver); (b) the data server, where all captured image data is finally stored for persistence and post-processing; and (c) the user, selecting adequate scanning parameters. Depending on the vendor, the acquired data is provided and stored in various types of common data formats such as (XML, STL, CSV, DICOM, mesh-types, meta-data) from which the user can freely select. As this interface is provided as part of the device itself, we consider it as an implicit part of the sensor. Nevertheless, it is worth mentioning that some post-processing possibilities described later on in “3D post-processing” section are sometimes already included in these interface packages and can hence directly be used and applied to the collected data.

### 3D data

3D data post-processing and conditioning is necessary to prepare the scanned and reconstructed raw data voxels, point clouds, and meshes for the successive additive manufacturing step. The discussed imaging modalities (see “3D data acquisition possibilities for the outer ear” section) yield different types of output data, which then need to be processed and converted, so they can be used for additive manufacturing (see “3D printing” section). A 3D mesh is the structural build of a 3D point cloud model, whose points are connected polygons. Polygons consist of triangles or quadrangles. The point clouds and related meshes define shapes with height, width, and depth. The source data of such meshes can be the reconstructed 3D point clouds from the optical scanning devices, where sets of local adjacent 3D-points are connected via triangles or quadrangles. Similarly, the outer surface of volumetric data (from CT or MRI) can easily be converted to a surface mesh using standard methods, such as marching cubes [72].

### 3D post-processing

Commercial software packages, such as ZBrush, 3D Studio MAX, Maya, AutoDesk Fusion 360, etc., offer access to the 3D point-clouds and meshes with various post-processing options (https://all3dp.com/1/best-free-3d-modeling-software/). A wide range of open-source solutions also exists for point-cloud visualization and manipulation, e.g., Blender 3D and MeshLab. Open source software applications can provide cost effective solutions for pre-clinical research applications^1^ [73–75]. These freely available software packages offer a wide range of image processing and point-cloud manipulation methods. Nevertheless, most of the possibilities and functions available are not needed for data pre-processing tasks.

Basic editing and manipulation tasks include data smoothing and outlier removal, as well as cutting, resizing, cropping, mirroring, and rotating along any of the axes. Additionally, filling holes (see example of missing *concha* in Fig. 5, right) and providing a certain minimum of thickness to the mesh are essential steps to establish printer friendly production files.

In the case of 3D data, such as point-clouds, the graphical user interface, the navigation and manipulation of the 3D-points, and the 3D mesh is different from 2D images. However, intuitively designed user interfaces along with 2D and 3D mouse handling allows for rapid learning and adaptation to the graphical interface. Furthermore, standard file handling commands (open, save) and the importing possibilities of different file formats (i.e., OBJ, PLY and STL) allow compatibility with most of the commercially available 3D-printers [76–78].

### 3D data registration

Besides 3D data post-processing (see “3D post-processing” section), two or more sets of 3D data – ideally obtained from of the same patient – can also be fused using image registration approaches. This type of image processing can, for example, be used if several scans of the same ear from different imaging devices are available in order to fill in missing information (such as the *concha*) or to compare the symmetry of both ears.

**Fig. 7:**
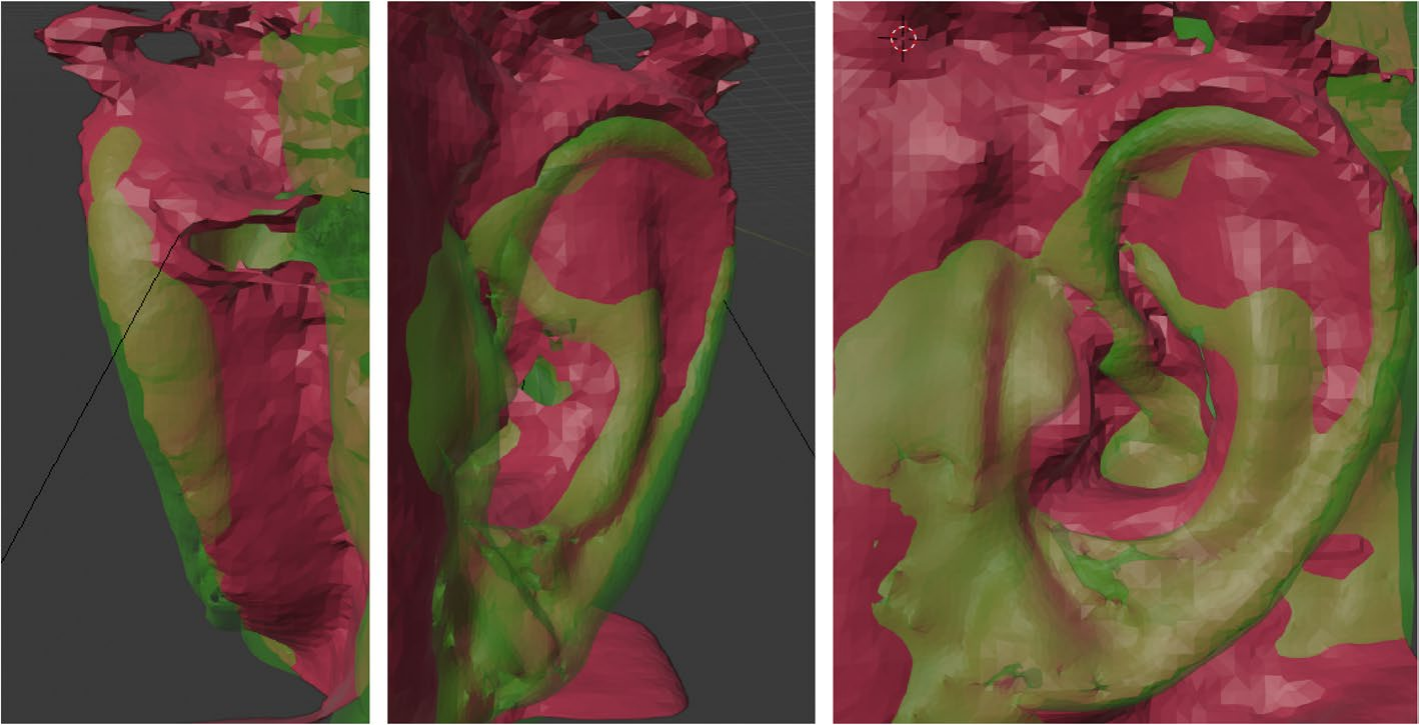
Image registration and image fusion of the MRI data (Fig. 3) and optical data (Fig. 6, left) obtained from same person (author TW). Left side: posterior-anterior view; center: anterior-posterior view; right side: side-view. ‘Brown’ indicates data from the optical scanner, while ‘green’ represents data from the MRI. The MRI data has a much higher resolution quality and is much smoother than the data obtained from the optical scanner

In Fig. 7 an example of such image registration of the optical data (Fig. 6, left) (depicted in ‘brown’) and the corresponding MRI data (Fig. 3) (depicted in ‘green’) of the same left outer ear is provided. In this figure the views from left to right are: posterior-anterior view, anterior-posterior view, and side-view. Here, the MRI data (green) has a much higher resolution quality and is much smoother than the data obtained from the optical scanner (brown).

**Fig. 8:**
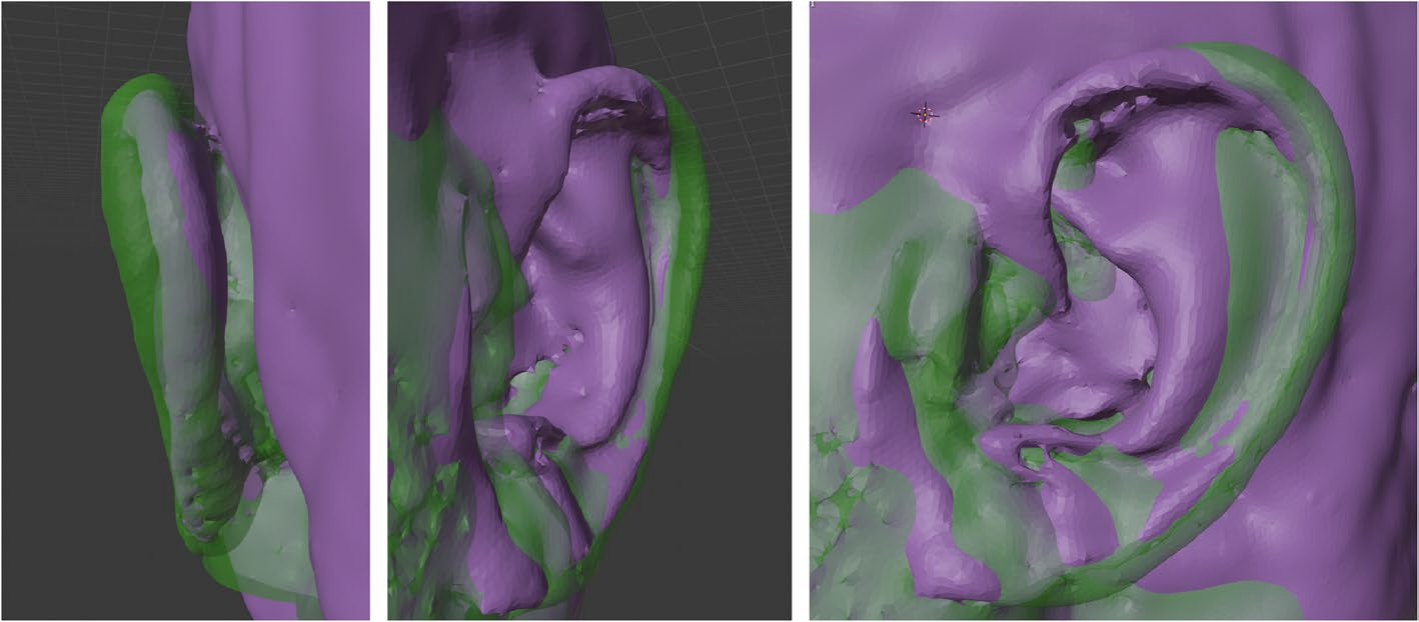
Image registration fusion of left and right outer ear from the MRI data (Fig. 3). Left side: posterior-anterior view; center: anterior-posterior view; right side: side-view. ‘Green’ represents data from the left ear, ‘purple’ indicates data from the right ear. Some asymmetries can be observed between the ears, which are rarely perceived in daily routine

In Fig. 8 an example for the image registration between the left and the (mirrored) right outer ear (from MRI) is provided. Left ear data is depicted in ‘green’, right ear data in ‘purple’. Similar to before, the views from left to right are: posterior-anterior view, anterior-posterior view, and side-view. Even though both outer ear data sets are from the same person, there is a visible asymmetry between the left and right outer ear.

### 3D shape models

Besides manually or interactively optimizing the acquired 3D data (see previous “3D post-processing” section) with respect to later additive manufacturing and printing, it can also be used to construct 2D- or 3D shape models of the human ear [79, 80]. These shape models are based on large scale collections of 2D or 3D image data of the pinna [81] in which a fixed and predefined set of prominent key points – or landmarks – on the outer ear structures (see Fig. 2) have been manually marked. For example, in the work by Dai et al. [79] 55 landmarks have been annotated manually on all ear data, namely along the outer and inner helix, the ear lobe, the tragus and antitragus, the concha, and ear canal as well as the inferio and supperio crus. Using these key points, a so-called statistical shape and appearance model can be obtained, whose Eigenvectors or variation of variances for each axis can be used to deform the shape model in any direction and shape. Even though in the literature [79, 80] such shape models of the ear are used for biometric characterization of subjects, within the addressed application for outer reconstruction such shape models could possibly be used as an enhancement for post-production, e.g., filling the missing parts of the 3D data.

## 3D printing

3D scanners or capturing devices can acquire details of the anatomy of the human body, including ears, nose, or even facial expressions [82–85]. A time and cost effective workflow pipeline for printing body parts - such as outer ears - in a rapid prototyping procedure, based on scanning, image processing, printing, and post-processing requires:

- a handheld mobile device (scanner or smartphone) with adequate (high) resolution, easy installation and usability;
- an (open-source) software solution for image processing with basic functionality;
- widely available 3D printers and/or customized desktop devices;
- a wide range of hard and/or soft elastic materials (avoiding expensive biomaterials);
- a set of printed prototypes for post-processing and finishing.

All these allow for individual adjustment to the patients’ needs. Finalizing the end product needs iteration steps where an inexpensive workflow plays a significant role.

3D printing is an additive manufacturing technology. In contrast to subtractive manufacturing, it does not require a block of material, but rather stacks and fuses layers of material. The first ISO/ASTM joint standards on additive manufacturing were published in 2013. Since then, many updates have been released, including the ISO/ASTM 52910 for product design and the latest version of the ISO/ASTM 52900 “Additive manufacturing - General principles - Fundamentals and vocabulary”, as the internationally recognized source for terms and definitions [86–88].

**Fig. 9:**
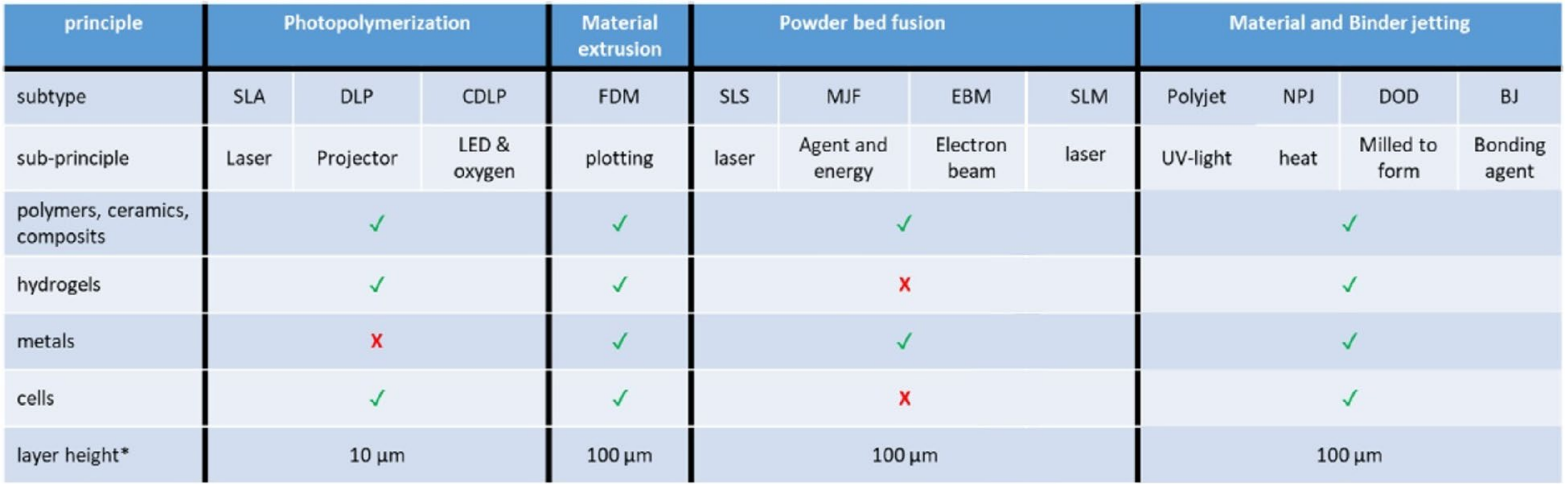
Overview of 3D printing methods, their methodological principles, sub methods, and selected characteristics of the respective method. SLA: stereolithography; DLP: Digital light processing; CDLP: Continuous digital light processing; FDM: fused deposition modeling; SLS: selective laser sintering; MJF: multi jet fusion; EBM: electron beam melting; NPJ: nanoparticle jetting; DOD: Drop on Demand; BJ: Binder jetting. *Layer height varies based on 3D printer and material, so the stated number is a rough guideline value. Green check mark: possible/good performance; red X: not possible/low performance

For biomedical application in general, different types of 3D printing can be categorized based on their manufacturing principle (Fig. 9). The most commonly used 3D printing process is photopolymerization, which includes stereolithography (SLA), digital light processing (DLP), and continuous digital light processing (CDLP). Using photopolymerisation, a wide selection of materials, high resolution, and high quality surface finishes are possible. During the photopolymerisation, light (laser, projector, LED) causes monomers and oligomers to form polymers in a layer-by-layer process [89–91]. Powder beds can be fused by laser (Selective Laser Sintering (SLS) or Selective Laser Melting (SLM)), agent and energy (MJF, Multi Jet Fusion) or electron beam (EBM, Electron Beam Melting). Material-extrusion-based additive manufacturing is also called fused deposition modeling (FDM). Although processes can be different based on materials, melting point, energy consumption, and type of beam, SLS, SLM, and EBM processes are very similar [92]. SLS operates below the melting point, while SLM operates above it, thus, the latter is less energy saving. EBM has advantages, as it is even more energyfriendly and provides a more uniform thermal field distribution. However, dimensional accuracy is lower. Figure 9 shows a list of printing methods and their characteristics.

All aforementioned 3D printing methods are generally suitable for producing reconstructions of the outer ear. However, there are economic differences depending on the chosen technique. Devices based on extrusion, in general, are more cost-effective than other methods but restricted in resolution. Also, when selecting the printing method, it must be noted that there are processes that are restricted to only one material, resulting in required post-processing for the removal of support structures (e.g., in the case of photopolymerization). Other technologies allow for the use of different materials within a single print job, enabling the generation of support structures from dissoluble materials, thus avoiding time-consuming post-processing. It is also necessary to consider the required resolution. For creating replicas of the outer ear, accuracy in the millimeter range is sufficient. Corresponding printer systems can already be obtained nowadays starting from €2000.

Replacing molding of silicone, urethane, and rubber parts can be achieved in a matter of hours using flexible and elastic resins. For hard flexible prototypes, a balance between softness and strength has to be maintained to withstand bending, flexing, and compression through repeated cycles. Soft flexible prototypes are mechanically similar to silicone parts. They also have to withstand mechanical impact and spring back quickly to their original shape. Rigid polyurethane materials can be also used when long-term durability and skin contact is required. Printing parts directly is a main contributor to time and cost savings [33, 93].

3D produced parts also require post-processing, i.e., washing, rinsing, streamlining, and surface finishing. Some of these procedures can be automated, but many of them still have to be made by hand. In particular, the post-sculpting of the replicas to adapt them to the individual needs of the patient extends the manufacturing time. In the case of ear replicas, if the mirrored image of the contralateral side of the head is used, asymmetries will not allow direct application of the printed part. Furthermore, accuracy of printed organs with complex geometry depends and varies on the 3D printer model used. It is possible to improve the controller algorithm for better results [94].

### Biomaterials

Biomaterials are designed for professional healthcare applications that require medical-grade materials for biocompatibility. Such materials are usually produced in certified facilities, and are compatible with sterilization and disinfection methods. Parts are suited for long-term skin or short-term mucosal membrane contact (tissue, bone), and are also suitable for pharmaceutical applications.

Bioprinters are capable of printing with biologic inks, a method that is most advanced in high quality tissue engineering. The ink must be stiff enough to capture the topography of the auricle. The use of support materials, which can be later removed, could be also a good solution. A good overview of the evolution of tissue engineering methods can be found in [95].

Printable bioink was constructed based on goat ear cells for desired viscosity and polymerization, and a pinna was printed for biocompatibility testing [96, 97]. Although the patented procedure was found to be successful for transplanting ear replicas, creating the biocompatible ink was time consuming and circumstantial.

Biological cells grown using additive manufacturing can also be used with electronics. A bionic ear was printed together with conducting polymer, where an inductive coil antenna was integrated inside the ear cartilage for radio frequency communication that allowed for stereo music listening [98].

Printing functional organs with bioprinting is still difficult, as functionality has to be maintained during the integration of different cells [99]. Repairing outer ear defects with cartilage tissue does not need to be fully vascularized to function, thus, by avoiding using bioinks, functionality can still be restored by printing simple synthetic materials.

### Synthetic materials

3D printing without biomaterials can also produce replicas for reconstruction interventions and surgery using various elastic materials. A mirrored CT image of a healthy ear was used for 3D modeling and printing using polycaprolactone or silicon was used to overcome the limitations of previous auricular reconstruction methods [100, 101]. However, the authors have highlighted the need for further studies to extend the clinical use of 3D-printed constructs.

A complete protocol was developed for FDM printing using two different materials to address the challenge of reconstructing stick-out ears [102]. This method applied the steps of scanning, reverse engineering, individual prototype design, and printing, focusing on the mechanical properties and preservation of the auricular shape.

A systematic review of maxillofacial prosthetics included nasal, auricular, and ocular prostheses [103]. Auricular methods preferred laser scanning and different CAD methods for mirroring the healthy ear. Moreover, in the case of bilateral damage, a digital library was proposed for a selection of available scans. The review covered papers from 1992 to 2019 and, in this period, the printing of a mold and creating replicas traditionally were preferred due to issues of color matching and fit of the replicas (indirect prototyping). In the case of external ears, functional movement and moisture of the replica were not critical issues, thus a wider selection of materials could be used. Direct prototyping reduces time and costs, however, indirectly printed molds can be used later for replacing missing or deteriorated parts more easily. Molds can be designed as a negative volume around the scanned part, followed by division into multiple sub elements. The pinna is not optimal for mold print methods because of the shape. Certain volumes and curvatures cannot be printed without a support that has to be removed later, holes for filling have to be designed, and cutting and removing of the mold would need high accuracy. Another current review examined 3D printing methods in otolaryngology surgery [104]. It states that although the use of technology is constantly growing, however, possibilities are still limited. In conclusion, further case-control studies and long-term analyses were suggested to evaluate all benefits of this technology.

**Fig. 10:**
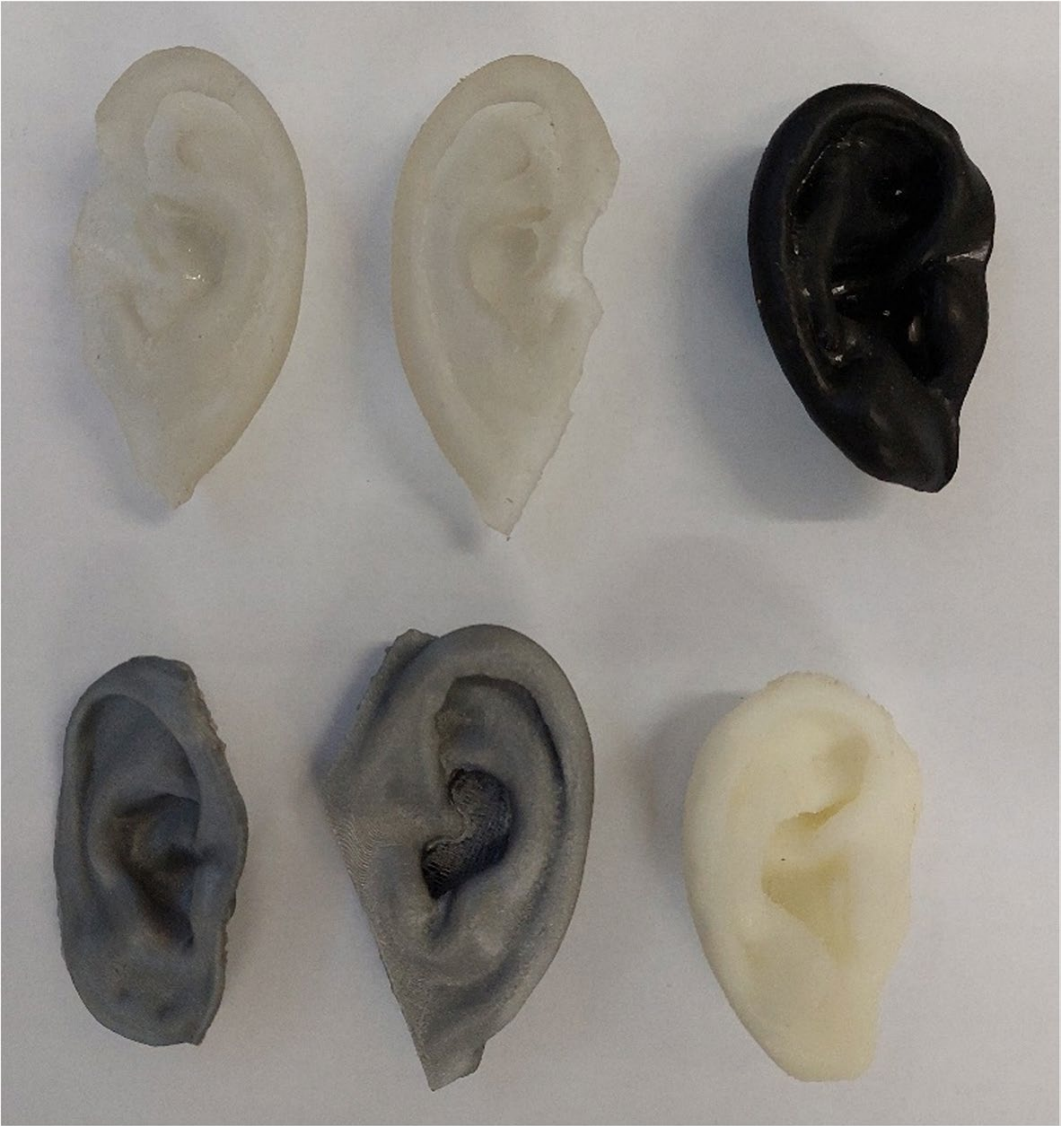
Examples of printed replicas using hard polymer and synthetic resin. The top left is the mirrored version of the top middle replica

**Fig. 11:**
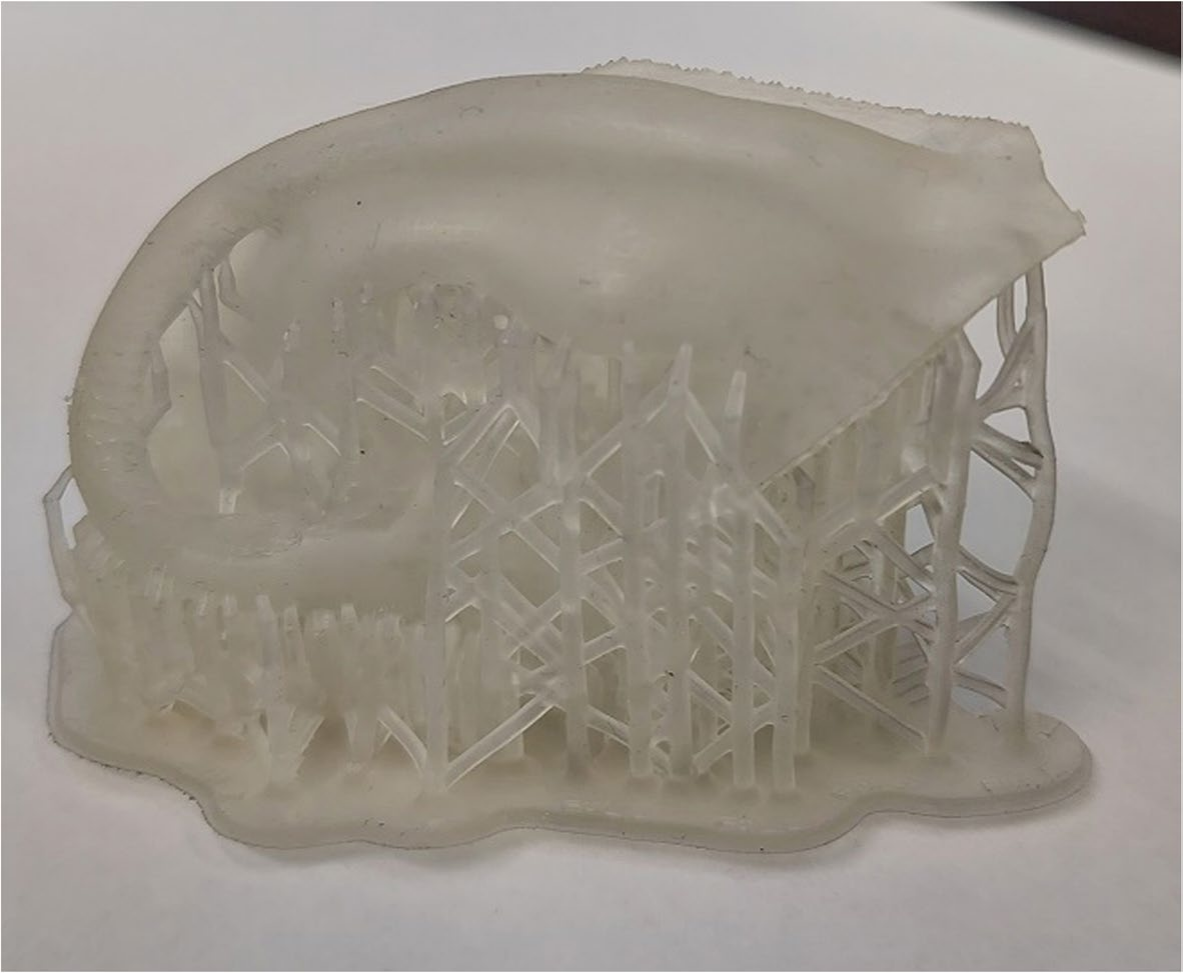
Printed ear with supporting material

Figure 10 shows various outer ear replicas. After printing, polishing the prints, removal of residual material and surface unevenness were required. Direct printing of external ears needs supporting material, such as pillars that have to be removed (Fig. 11). Alternatively, water-soluble support can be printed and dissolved in water.

External ear components (base plate, helix, antihelix) were printed with silicone, colored, and assembled by trainees during an ear reconstruction workshop [105]. Participants found this technique advantageous and more efficient than traditional time consuming and technically demanding auricular reconstruction procedures.

Prototype ear replicas of elastic materials were also tested for usability and compatibility with everyday use, including tensile testing for water and chemical resistance (hot and cold water, soap, shampoo, after shave), high and low temperatures (boiling water vapor, freezing), without any detectable damage [106]. A former experiment found conventionally packed silicone to be more resistant than directly printed samples [107].

3D printing of external ear replicas using synthetic materials decreases surgery time due to reducing the need for hand carving processes, while increasing precision and accuracy. However, post-sculpting and painting of the replicas to match skin color is still an issue. Combined multi-material printing using elastic materials can mimic both tactile feel and skin pigmentation [108].

## Future perspectives

### Validation

**Fig. 12:**
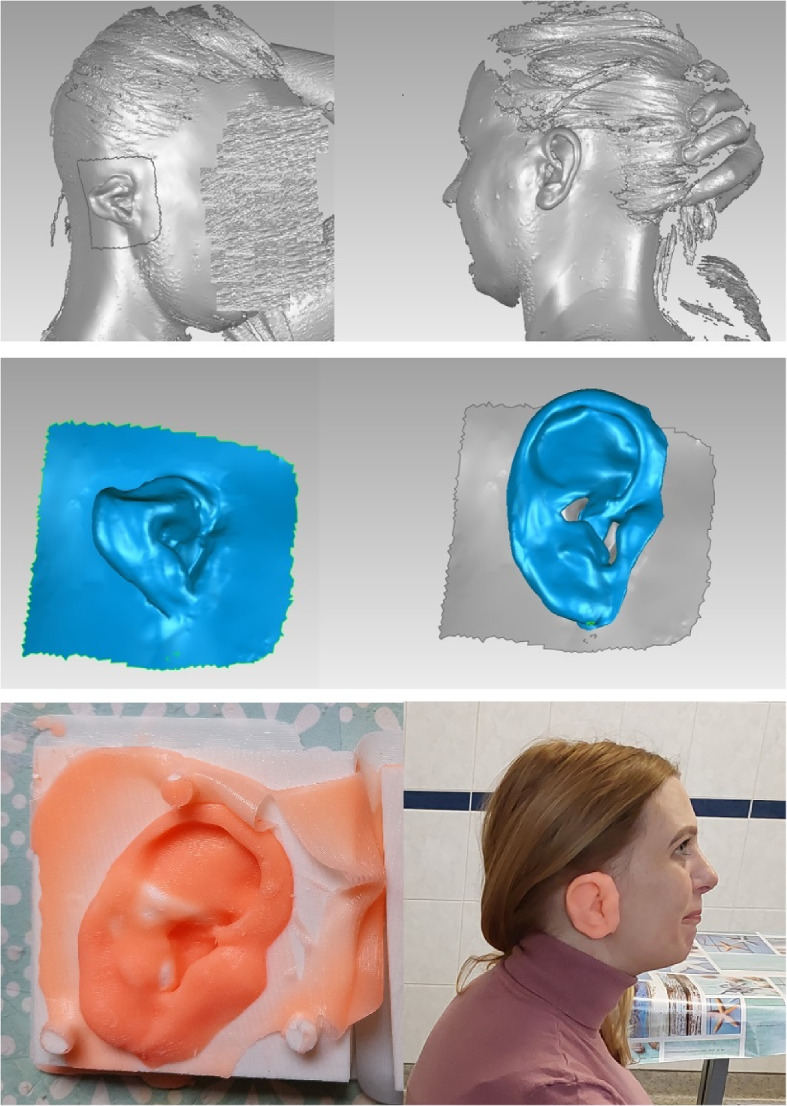
Reconstructed right ear replica based on the mirrored image of the left side. Image was scanned with a handheld scanner and post-processed in MeshLab. A series of prototypes were created using a mold and tried on in an iteration process. Fixation methods can be tested after the final model is printed

In order to validate the workflow pipeline, the process was demonstrated in a clinical example with a 30-year-old female patient, including scanning with a middle-range device (Creaform Go!Scan), post-processing in MeshLab (mirroring of the healthy ear and digital sculpting), and manufacturing a series of replicas using a mold and elastic material (Fig. 12).

In a workflow process, 3D printers are the most expensive part, thus making printing more economical and easier is important. Patient-customized ears were constructed in solid form and with limited accuracy in porous form by means of a modified low-cost desktop printer [109]. The dimensions and quality were sufficient for the selected tissue engineering applications. The same printer (Ultimaker 2+) was also selected for its low-cost multiscale templates to prove that there were no structural changes to sterilization [110]. 3D scans were used in a workflow pipeline to print 3-part negative molds with a low-cost desktop 3D printer; they were then casted with silicone to produce ear prostheses [111]. This production framework proved to be an effective and alternative method to current techniques. In-house production will also be available for tissue engineering in the future.

Microtia, an underdeveloped external ear, is a common congenital malformation occurring in approximately 0.03% of births [112]. The malformation can affect size, orientation, shape, and location of the auricle, additionally, autologous costal cartilage has proven to be the most reliable technique for building an ear framework [82]. To date, total pinna reconstruction based on 2D or 3D images of the unaffected contralateral side is the best treatment for congenital microtia [101, 113–115]. However, the process of imaging, reconstruction, framework set up, and surgical intervention is a difficult and cost intensive process.

Traditional methods for microtia reconstruction include collecting cartilage from ribs then sculpting and assembling it into a three-dimensional ear scaffold before implantation [82, 95, 112, 116]. The result is not necessarily elastic and the procedure is painful. Methods can be one-stage or typically multi-stage (four-stage Brent, two-stage Nagata) and all have advantages and pitfalls [117, 118]. The number of stages refers to the number of operations needed to reach the final outcome. The classification and management of skin samples for the rib cartilage are key steps during sculpting and finalizing the product [119, 120]. Autologous reconstruction methods are highly durable with low infection rates, but may need multiple operations and are accompanied by pain. In contrast, tissue engineering operates without donors at a minimal infection rate, but is expensive [121].

In the case that there are no healthy ears available for scanning, public databases can be accessed. These databases offer 3D models of the outer ears, i.e., the York Ear Model database [122], the OpenHear database [123], the Bulgarian ear database [124], the AMI Ear Database (https://webctim.ulpgc.es/research_works/ami_ear_database/), the UBEAR dataset [125], the University of Notre Dame databases (https://cvrl.nd.edu/projects/data/#nd-collection-j2), and the SYMARE database (https://pure.york.ac.uk/portal/en/datasets/sydney-york-morphological-and-recording-of-ears-database-symare).

Fixing methods of external ear prostheses include applying prosthesis glue, magnets or “piercing” methods. It is also possible to omit any chemical or mechanical fixation. Replicas may be removed accidentally or on purpose, e.g., during sleep or exhaustive body exercise. Larger replicas, e.g., a whole pinna, may be fixed permanently by applying screws, stitches or magnetic fixations with surgical intervention.

### Acoustics and psychoacoustics

The external ear has been shown experimentally to contribute enormously to localization in human spatial hearing [126–128]. In particular, the individual shape of the pinna causes directional and frequency-dependent filtering of incident sounds, thus contributing to the decoding of directional information from sound sources. Together with the head and torso, the pinna is responsible for the so-called Head-Related Transfer Function (HRTF), i.e., an acoustic function used to characterize sounds arriving from various locations in space [129, 130]. The HRTF is highly individual, as a matter of fact, since each person has a different anthropometry, it follows that each person has a different HRTF. HRTFs can be acoustically measured using dummy heads or human subjects and they can be used for virtual sound source simulation (auralization) [131].

By analyzing unique acoustic cues humans are able to localize sounds in vertical space, although vertical localization has lower resolution than horizontal localization, where interaural cues play a major role [132]. The understanding of the spectral cues responsible for vertical localization was enhanced by a number of investigations [133–138]. Particularly, Hebrank and Wright proved that spectral cues for vertical localization occur between 4 and 16 kHz and that a sound must be located within this frequency range to be localized vertically. These cues, which take the shape of spectral peaks and notches, produced by acoustic processes of resonance, reflection, and diffraction are known to be produced by the pinna.

It has been shown that the progressive occlusion of the pinna cavities has the effect of severely degrading vertical localization with noticeable effects especially at high frequencies [139]. Therefore, preserving the whole shape of the pinna is essential to the localization accuracy of human subjects. This means that an injured, deformed or missing pinna results in decreased localization performance. Reproduction and reconstruction of damaged outer ears not only contribute to aesthetics, but also to functionality in spatial hearing. Especially in case of newborns and children, it is important to restore functionality besides aesthetics [140]. When replicas have to be replaced as the head and body parts grow, simple and cost effective solutions are required.

The effect of having new ears on sound localization has been investigated over the past 30 years; however, designing such experiments is difficult [141, 142]. One way is to repeat the same listening test before and after the process to check whether any variation in localization can be detected. Nowadays, 3D technology allows for scanning and 3D printing of not only outer ears [143], but also of the geometry of the whole head for the same purpose [144–147]. In this case, HRTFs can be measured using binaural microphones placed at the entrance of the ear canal of the printed head. Nevertheless, evaluating differences and variations in HRTFs does not directly imply an evaluation of localization performances for which subjective listening tests are required.

### Outlook

A majority of current experiments in the field of additive manufacturing focus on individualization and personal health care. The main problem during development in the medical field is the highly regulated market, which also imposes highly regulated conditions on experimental processes. Medical-grade equipment and materials have to be approved, used in a suitable manner, and manufactured to certain standards. On the other hand, regulation of emerging technologies is underdeveloped.

In the case of tissue engineering and related fields, besides fundamental philosophical and bioethical questions, issues of practical risk, biosafety, and security have to be addressed. Conventional biomedical products have established regulatory pathways, and well-defined classifications and standards. Material properties are defined, and provided by qualified suppliers with known risks and certifications. In clinical trials standards apply. For emerging technologies, international standard orders, drafts, and guidelines have been released that are less defined. This field is only partly regulated; standards can be adapted with limitations. Risks are higher based on the patient-customized nature of the process. Current standards are more suited for conventional therapeutics and in the future regulatory authorities have to learn the background and complexity of the advanced products. An overview on the current legislations and standards (with a special focus on 3D bioprinted tissues), challenges in the clinical applications, ethical concerns, and future directions are discussed in detail in [148, 149].

Applied materials range from composites, polymers, ceramics to bioematerials [150]. Significant progress is expected using bioprinted human tissue models to accelerate drug discovery and development. Tissue bioprinting techniques, such as inkjet printing, laser-assisted printing, extrusion, and cell electrospinning will be applied to produce tissues from individual cells to produce complex tissues and organs [151]. The outer ear and/or nose could be the first step in the direction of using 3D technology expertly.

A promising future direction is direct 3D printing of silicone. Silicone is a synthetic rubber with a versatile chemical structure. It has high thermal stability, resistance against oxidation, compression, water, and UV light; moreover, it can be sterilized (biocomaptibility), and can have different hardness, flexibility, and color options. Currently, most products are produced with injection or compression molding or casting. Direct 3D printing would remove the model and mold steps by printing the final product, thus, saving cost and time. Silicone has a high viscosity making it difficult to print directly in 3D. Furthermore, it cannot be cured or heated with UV light. Specialized printers are still rarely accessible and expensive for everyday business. Printers can not print with exactly the same material that is used during injection molding, as accuracy, detail, and material options are limited. Liquid additive manufacturing (LAM) and silicone additive manufacturing (SAM) are emerging fields where silicone is made to be light-sensitive in order to be cured. Silicon, once solidified, can not be made elastic again. It needs additives to be sensitive to light or heat, but this can weaken the material properties and affect lifespan. Dissolvable support structures can be used, but it has to be removed, and additional curing or vulcanization may be required on the final product. Available printers on the market are expensive and they are used for creating low-volume parts, i.e., custom-fit wearables, hearing aids, and earphones, even with complex internal geometries. Outer ear replicas could be an extension to the possibilities. A current overview of available solutions can be found in (https://all3dp.com/2/silicone-3d-printer-all-you-need-to-know/).

The growing market for machine and material providers will lead to reduced machine costs, and increased print speed will additionally force prices down. Integrated solutions - such as presented in this paper - can be sold, i.e., scanners could be shipped as a part of the printer itself. Healthcare service providers have to develop innovative services, which will differentiate themselves from competitors by creating business models with hospitals. Additive manufacturing will also drive sustainability forward. Benefits can far outweigh costs both for hospitals as well as for patients. Currently, leading applications are in orthopaedics, followed by cranio-maxillofacial, neurosurgeries, and cardiology.

## Conclusion

This paper reviewed current trends in the production of cost effective replicas of the human outer ear. Commercially available 3D handheld scanners, open-source image processing applications, and 3D printing based on synthetic materials allow for cost and time effective rapid prototyping of prostheses for everyday use. Alternatively to the time and cost of expensive tissue engineering methods, individually personalized replicas can be designed and fabricated for reconstruction of damaged and injured ears. Moreover, using polymers solves the ethical and legal problems regarding the growing and transplanting of human or animal cell cultures. Simplified workflow pipelines allow doctors to reconstruct outer ears in order to improve a patient’s well-being, aesthetics, and functionality.

## Data Availability

Not applicable.

## Appendix A

**Table 1:** Overview and comparison of selected devices. Similar models may be available on the evergrowing market

**Device**	**Creaform Go!Scan**	i**REAL 2S**	**SIMSCAN**	**EinScan H**
**color**	yes	yes	yes	yes
**accuracy (mm)**	0.05	$3-0.2$	0.02	0.05
**speed (measurements/second)**	1.5 million	1.5 million	2.02 million	1.2 million
**method**	white light scanning lines	blue LED light and infrared light	crossed blue lasers	hybrid LED and infrared light
**mesh resolution (mm)**	0.2	0.1-0.15	0.02	0.25-0.3
**price (EUR)**	36.000	12.000	30.000	6.000
**Device**	**RevoPoint**	**Creality CR-Scan Lizard**	**SENSE 3D**	**iPhone13 cellphone**
**color**	yes	yes	yes	yes
**accuracy (mm)**	0.02 - 0.3	0.05	0.3 - 1	range dependent
**speed (frame per second)**	10-18 fps	10 fps	10 fps	n.a.
**method**	Dual-camera infrared or blue structured light	LED and NIR (near-IR) light	Dual-Camera Infrared Structured Light	Built-in 2D optical camera of 12 Mpixel
**mesh resolution (mm)**	$0.05 - 0.3$	0.1 - 0.2	0.5 - 1	n.a.
**price (EUR)**	700	700	350-650	1000

## Footnotes

1 For clinical applications the applied software needs MDR/FDA approval.
